# The Predictive Role of Plasma Biomarkers in the Evolution of Aortopathies Associated with Congenital Heart Malformations

**DOI:** 10.3390/ijms23094993

**Published:** 2022-04-30

**Authors:** Amalia Făgărășan, Maria Oana Săsăran

**Affiliations:** 1 Department of Pediatrics III, Faculty of Medicine, George Emil Palade University of Medicine, Pharmacy, Science, and Technology of Targu Mures, 540136 Târgu Mureș, Romania; amalia_fagarasan@yahoo.com; 2 Department of Pediatrics III, Faculty of Medicine in English, George Emil Palade University of Medicine, Pharmacy, Science, and Technology of Targu Mures, 540136 Târgu Mureș, Romania

**Keywords:** aortopathy, plasma biomarker, congenital heart disease, acute aortic dissection, matrix metalloproteinase

## Abstract

Dilatation of the aorta is a constantly evolving condition that can lead to the ultimate life-threatening event, acute aortic dissection. Recent research has tried to identify quantifiable biomarkers, with both diagnostic and prognostic roles in different aortopathies. Most studies have focused on the bicuspid aortic valve, the most frequent congenital heart disease (CHD), and majorly evolved around matrix metalloproteinases (MMPs). Other candidate biomarkers, such as asymmetric dimethylarginine, soluble receptor for advanced glycation end-products or transforming growth factor beta have also gained a lot of attention recently. Most of the aortic anomalies and dilatation-related studies have reported expression variation of tissular biomarkers. The ultimate goal remains, though, the identification of biomarkers among the serum plasma, with the upregulation of circulating MMP-1, MMP-2, MMP-9, tissue inhibitor of metalloproteinase-1 (TIMP-1), asymmetric dimethylarginine (ADMA), soluble receptor for advanced glycation end-products (sRAGE) and transforming growth factor beta (TGF-β) being reported in association to several aortopathies and related complications in recent research. These molecules are apparently quantifiable from the early ages and have been linked to several CHDs and hereditary aortopathies. Pediatric data on the matter is still limited, and further studies are warranted to elucidate the role of plasmatic biomarkers in the long term follow-up of potentially evolving congenital aortopathies.

## 1. Introduction

Aortopathies or the dilatation of the aorta involving the root or descending aorta have gained a lot of interest recently due to the significant progress in imaging techniques, which has led to a greater understanding of their pathophysiological underlying mechanisms [1,2]. Traditionally, these conditions are most frequently associated with congenital heart disease (CHD) but also comprise of hereditary aortopathies encountered among genetic syndromes such as Marfan, Turner or Ehlers-Danlos [2]. Thus, their classification is based upon three main etiological subtypes, namely [3,4,5,6,7,8]:

- Syndromic—associated with genetic syndromes (Marfan, Loeys-Dietz, Ehler-Danlos type IV and Turner) and presenting an increased risk of dissection;
- Primary—associated with CHD, including aortic coarctation, conotruncal abnormalities (persistent truncus arterious, great vessels transposition, double outlet right ventricle, tetralogy of Fallot, double outlet right ventricle), left heart hypoplastic syndrome or bicuspid aortic valve (BAV);
- Secondary—a consequence of surgical intervention (arterial switch, ROSS or Fontan procedure).

In adults, the dilatation of the aorta is defined as dilation that exceeds 1.1–1.5 times the normal caliber, whereas an aneurysmal dilation is even greater than 1.5 times, with normal reference values established through wide-based population studies [9]. However, in children, the assessment of aortic dilatation is based upon the z score, with an upper limit of 2.1 being taken into account as an absolute increase in aortic diameter. This z score is based upon sex, height, weight, age and body weight surface and is considered a more reliable indicator over time that is able to assess dissection risk [10]. These measurements of the aorta can be easily performed with the help of transthoracic echocardiography (TTE). Regular 2D ultrasound imaging of the aorta is mandatory, not only for diagnosis but also for follow-up, in order to assess the evolution of the aortic caliber and to establish the timing of prophylactical surgical intervention for preventing the ultimate life-threatening event, aortic dissection. Its main limitation is the underestimation of the largest caliber of the vessel lying in the lateral plane in subjects with asymmetrical aortic dilatation [10]. Therefore, a more precise assessment requires multimodal imaging [11]. This includes 3D ultrasound, as well as transesophageal echocardiography (TOE), which allows the visualization of the aortic valve, root and ascending and descending aorta. Furthermore, 3D TOE is apparently able to better evaluate entry tear size, but its additional utility over 2D TOE has yet to be established [12,13]. TOE allows measurements similar to those provided by computed tomography (CT) or magnetic resonance imaging (MRI) and is especially preferred in those subjects in which the administration of contrast substance is put into question (due to a history of allergies or renal function impairment), but this is highly dependent upon the examinator’s experience [11]. In particular, CT, consisting of non-enhanced scanning, followed by a contrast-enhanced angiography, presents the advantage of quick image acquisition and 3D reconstruction, but ionizing radiation exposure associated with its use limits its application as a follow-up instrument, especially in children [14]. The main drawback of the aforementioned imaging tools remains their uneven availability and their dependence upon their performance techniques [15,16,17].

The physiopathological mechanism description of aortopathies is strongly based upon the embryological development of the cardiac structure and the adaptative structural modification of the aortic wall, which are initiated during fetal life and continue in the first postnatal weeks. The main histological components of the great vessel wall, elastin, collagen and smooth muscle cells (SMCs), undergo significant changes due to local hemodynamic alterations. For example, the last pre-natal week sees a rapid accumulation of collagen and elastin in the wall of the ascending aorta, particularly involving its thoracic component [17]. Still, data in the literature supports the theory that blood flow or pressure variation determines continuous alterations in smooth muscle and collagen contents. This process will continue after neonatal life, whereas elastin expression will lower significantly after birth, and thus elastin content will remain stable through most of life [18,19].

A better understanding of the pathogenesis and of the continuous evolvement of aortic dilatation has created the premises for several studies that have tried to identify biomarkers with the ability to predict changes in the structure of the aortic wall and to distinguish etiological subtypes [20,21,22]. Most of them have evolved around the role of proteolytic enzymes, which determine a reduction in the extracellular matrix, thus leading to an abnormal remodeling of the aortic wall media. Special attention has been given to metalloproteinases, as well as to tissue inhibitors of metalloproteinase and the impact of their disbalanced secretion [20]. Other examples include asymmetric dimethylarginine (ADMA), the soluble receptor for advanced glycation end-products (sRAGE) and transforming growth factor-β1 (TGF-β1) [23,24,25]. Protein units such as hemoglobin subunits alpha, beta and delta have been positively correlated with the maximal ascending aortic diameter, whereas mannan-binding lectin serine protease has been negatively correlated with the same parameter [21]. The ultimate goal of recent research is to investigate the utility of biomarkers, which can be quantified in serum plasma, but studies are still undergoing, and most of the research has so far focused on the bicuspid aortic valve due to its high frequency among the general population [26,27,28,29,30].

This review aims to highlight the diagnostic and prognostic role of the most intensely studied serum plasma biomarkers in different aortopathies, in light of recent literature data. A special focus will be given to pediatric aortic dilatation, which is an evolving condition that requires close and continuous follow-up.

### 1.1. Matrix Metalloproteinases (MMPs)

MMPs are a family of proteolytic enzymes that cleave different components of the extracellular matrix. Their activity is highly dependent upon the extracellular matrix components, and their expression is rather low in healthy tissues. The homeostasis of the vascular wall is insured by the balance between MMP, plasminogen activator–plasmin system activity and the concentration of tissue inhibitors of metalloproteinases (TIMPs) [31,32]. MMP expression is regulated by several growth factors and cytokines that upregulate or downregulate the transcription of their genes [33]. The in vivo function of MMPs still requires elucidation, as most of these are secreted as inactive zymogens and can only be detected by zymography, but enzymatic activity has been linked to their gene expression [34,35]. Therefore, the discovery of their activators, such as membrane-type matrix metalloproteinases (MT-MMPs), represented an important step towards understanding MMP role, as they are bound to the cell surface and regulate cellular function [34].

Evidence has emerged regarding the central role of MMPs in vascular remodeling, with studies supporting an association between their increased expression and atherosclerosis progression through the migration stimulation of smooth muscle cells towards the intima of the vascular wall [36,37]. An emphasis on these two previously mentioned metalloproteinases (MMP-2 and MMP-9) in recent studies can be attributed to their critical, proven roles in aneurysm development. MMP-2 is found in mesenchymal cells and seems to be linked to macrophage invasion, being the highest abundant elastase in abdominal aortic aneurysm tissue [38]. MT-MMP-1 represents a major activator of pro-MMP-2; the inactive precursor of MMP-2 and its inhibition in the context of shear wall stress has been associated with reduced MMP-2 secretion and the invasion of smooth muscle cells through the extracellular matrix [39,40]. MMP-9 degrades extracellular components of the aortic wall, being expressed by smooth muscle cells, lymphocytes and macrophages. Its activation seems to be triggered by toll-like receptor 4 (TLR4), which can be produced by vascular SMCs and mediates inflammation processes. Both MMP9 and TLR4 seem to be overexpressed in patients with abdominal aortic aneurysms and seem to be together involved in the pathogenesis of aortic remodeling [41]. In a similar fashion to MMP-2, an increase in MMP-9 levels and balance change MMP-9/TIMP-1 has been associated with valve remodeling [42]. This process is also intensely linked to ECM component alteration, in a similar fashion to the pathophysiological basis of atherosclerosis, especially in relation to the development of aortic stenosis and, to a lesser extent, to the one of aortic regurgitation [43]. Moreover, experimental studies conducted on mice showed that both MMP-2 and MMP-9 act complementarily in aortic aneurysm development [44]. MMP-2 still remains, though, the easiest to identify among mesenchymal cells, MMP-9 along with other MMPs such as MMP-3 and MMP-7 being synthetized by inflammatory cells [45].

Most of the literature data so far is based upon histology, and more precisely upon the immunohistochemical assessment of various MMPs within the aortic wall. A significant increase in MMP-2 and MMP-9 was noted by Koullias et al. in the bicuspid aortic valve in a study that enrolled patients with aortic stenosis with/without an associated aortic aneurysm/dissection [42]. In the same study, in the study subgroup of patients with an aortic aneurysm/dissection (seven patients with a bicuspid aortic valve and six patients with a tricuspid aortic valve), only a significant augmentation of MMP-9 expression was noted. Furthermore, TIMP-1 expression was higher in patients with a bicuspid aortic valve and aortic stenosis as opposed to their tricuspid valve homologues [42]. The best situs for determining the expression of each of the three aforementioned metalloproteinases (MMP-1, MMP-2 and MMP-9) seems to be the intima, where their overexpression can be noted at the entry dissection site of the ascending aorta, according to Ishii et al. [46]. Immunohistochemical studies have also highlighted differences in MMP expression within the abdominal aorta. MMP-9 seems to be produced to a higher extent in abdominal than in thoracic aortic aneurysms [47]. Furthermore, the expression of the mRNA transcript of MMP-9 might also be influenced by aortic diameters. In particular, a study that analyzed infrarenal aortic specimens proved that MMP-9 mRNA expression is higher in moderate diameter abdominal aortic aneurysms (5–6.9 cm) than in small ones, as they present better chances of further expansion [48].

The imminent, progressive evolution of aortic dilatation towards dissection calls though for the discovery and validation of circulating biomarkers, with an anticipatory role of catastrophic adverse events. MMP-1, TIMP-1 and MMP-2 were documented as reliable plasmatic biomarkers for the prediction of aortic surgery, positively associated with peak systolic wall shear stress (WSS) and time-average WSS (TAWSS). An increase in their circulating levels, in combination with non-invasively obtained wall shear stress parameters (with the help of 4D flow MRI and computed tomography angiography), seems to be highly sensitive and a specific marker of future ascending aortic aneurysm surgery [40]. Another study confirmed the upregulation of MMP-1 and MMP-2 in aortic dissection through a comparison with healthy controls, as well as the important role of miRNA-320 in their post-transcriptional regulation, after the lipopolysaccharide-induced activation of macrophages [49]. Nevertheless, MMP-9 has been documented as a reliable plasmatic marker of the final stages of aneurysm development processes in subjects with chronic thoracic aortic disease [50].

Given the high frequency of BAV among the general population, with an incidence reported as high as 2% [51], it is unsurprising that most of the research gravitating around the subject of MMPs has focused on the role of these biomarkers in the pathogenesis of complications related to this particular cardiac malformation. In BAV, a key element of aortic aneurysm pathogenesis is the imbalance between the proteolytic effect of MMP-2 and TIMP1. A comparative study conducted on subjects with aneurysms of the thoracic aorta, divided into two groups based upon the morphology of the aortic valve, proved that this particular disbalance is only associated with BAV, and not with the tricuspid aortic valve (TAV) [35]. Furthermore, in young men with BAV and the dilation of the proximal aorta, an increase in plasmatic levels of MMP-2 has been found, in association with systemic endothelial dysfunction [22]. This elevation in circulating MMP-2 level in association with aortic dilation in patients diagnosed with BAV, without any other echocardiographic abnormalities, has also been confirmed by another study, which reported simultaneously an increase in MMP-2 and MMP-9 circulating levels only in those patients with severe, isolated aortic stenosis [27].

Although most of the published research has focused on the role of MMP-1, MMP-2 and MMP-9 in the development of aortic pathologies, a few isolated studies have also addressed other MMPs as well. MMP-14 and MMP-19 showed higher mRNA expression in the media of dilated thoracic aortas of patients with TAV, with MMP-19 also being positively associated to maximal aortic diameters [52]. Ikonomidis et al. evaluated the implications of multiple MMPs (MMP-1, MMP-2, MMP-3, MMP-7, MMP-8, MMP-9, MMP-12, MMP-13, MMP-14 and MMP-15) and brought into light the paradoxical decrease in MMP-3 and MMP-14 in association with the medium-sized aneurysm group and BAV as opposed to the small or large aneurysm group. Contrarily, the authors reported a significant increase in MMP-7 levels in patients diagnosed with large aneurysms and TAV and an increase in MMP-13 values in patients diagnosed with medium-sized aneurysms and TAV [35]. Therefore, miscellaneous results and discrepancies between aneurysm size and aortic valve morphology underline the need for future studies that need to analyze the simultaneous implications of multiple MMPs in the pathogenesis of vicious aortic remodeling.

New insights into genetic polymorphisms of MMPs have been recently provided. Genetic polymorphisms of several MMPs, such as MMP-1, MMP-2, MMP-3, MMP-8 and MMP-12, have been associated with an increased risk of adverse cardiovascular events [53]. An increased risk of thoracic aortic disease was found in relation to genotype peculiarities of MMP-1 and MMP-9 in a study conducted in Poland [54]. Furthermore, a link between genetic polymorphisms of MMP-9 and TIMP-1 and the unfavorable evolution of abdominal aortic aneurysm repair has been documented through a positive association with endoleaks incidence [55]. Single nucleotide polymorphisms (SNPs) of MMP-9 have also been positively associated with both slow-growing and aggressively evolving abdominal aortic aneurysms [56]. Moreover, genotype particularities of MMP-2 have also been associated with aortic diameter increase and aortic dissection [57]. TIMP polymorphisms have also been shown to play an important role in the development of BAV aortopathy, with one study particularly showing that deleterious variants of TIMP-3 and hemizygous genotype of TIMP-1 pose an increased risk of developing this condition in patients with Turner syndrome [58].

Most research studies have so far enrolled adult and elderly patients, due to the evolving aortic dilatation in time and its related complications. MMP levels have been known to be positively associated with age increase and hypertension [50]. Still, the question of the utility of early MMP circulating values depiction has been raised, and thus pediatric studies on the subject have started to appear. Similarly to adult populations, a correlation between increased MMP-2 values and arterial stiffness has been reported in a pediatric population with chronic kidney disease and concomitant arterial hypertension [59]. Most pediatric studies have mainly focused, though, on patients with congenital heart defects. In children, a significant increase in serum plasma MMP-2 levels has been reported in association with Marfan syndrome, according to Cui et al. Contrariwise, MMP-9 presented insignificant variations compared to healthy controls within the same study [60]. Furthermore, another study accentuated the active implications of MMPs in connective tissue remodeling, proving an association between a significant increase in MMP-9 levels and the spontaneous closure of ventricular septal defects (VSDs) [61]. The authors also discussed the dependence of normal tissular and extracellular matrix (ECM) development upon MMP activity, as well as the insufficient data regarding the dynamic of MMP levels through the human heart and big vessel development [61]. Still, in subjects with congenital BAV malformations, the increase in MMP-2 and MMP-9 levels has been attributed to fibbrilin-1 deficiency among the vasculature, which seems to be independent of age progression and valvular function [62].

MMPs still remain the most researched entities in aortic-related pathologies and have so far shown the highest potential in evolving towards circulating biomarkers of this condition, based on recent literature data. A summary of the previously described studied and the association between tissular and circulating MMP expression variation, aortic anomalies and their complications has been provided in Table 1 and Table 2, respectively.

### 1.2. Asymmetric Dimethylarginine (ADMA)

ADMA represents an inhibitor of the nitric oxide synthetase, nitric oxide being responsible for active vascular remodeling. Nitric oxide (NO) regulates the endothelial secretion of pro-MMP-2, and elevated ADMA levels (which determine NO deficiency) will in turn lead to increased MMP-2 production [63,64]. Given the endothelial disfunction associated with BAV, some studies have successfully identified an association between elevated ADMA and MMP-2 and, consequently, the proximal dilatation of the ascending aorta in patients with non-stenotic BAV [28]. BAV morphology does not, on the other hand, necessarily influence ADMA levels, high ADMA values being associated with an ascending aortic diameter, without significant differences from patients with TAV [23]. The same lack of correlation with valve morphology, but with endothelial disfunction and inflammation mediator release, has been confirmed by Ali et al. [65].

A case-control study concluded that serum ADMA can be used as a reliable, highly sensitive and specific marker of ascending aortic dilatation [66]. Tzemos et al. further confirmed these findings, highlighting a positive association between the dilation of the proximal aorta and increase in serum ADMA and MMP-2 in a young male population diagnosed with BAV, as a result of systemic endothelial dysfunction [22]. Considering the increase in ADMA levels as a result of endothelial disfunction, literature data have proposed this biomarker as a tool that can assess cardiovascular risk [67]. This hypothesis started from oxidative stress, an essential element of the atherosclerotic process, increase in nitric oxide, leading to the stimulation of ADMA production, which will further inhibit the action of the nitric oxide synthetase [68]. Thus, ADMA has been regarded as a risk factor of acute coronary events, capable of inducing myocardial hypertrophy as well as fibrosis through the activation of fibroblast growth factors receptors [69,70].

In children, ADMA levels are physiologically higher, their decrease being associated with a lack of a protection against oxidative stress in conditions such as type I diabetes mellitus [71]. Their prognostic utility and potential use as a therapeutic target in pediatric chronic kidney disease has been described in a recent review article [72], but data regarding the biomarker role of ADMA in predicting the cardiovascular risk of this in patients is scarce. Furthermore, there are no pediatric studies in the literature on the subject of serum ADMA in aortic dilatation.

### 1.3. Soluble Receptor for Advanced Glycation End-Products (sRAGE)

The represents a receptor for advanced glycation end-products (AGEs), which are able to induce oxidative stress and trigger inflammation processes [73,74]. Furthermore, through ligand-binding competition for RAGE, sRAGEs attenuate the activation of the nuclear-factor kappa-B (NF-κB) pathway and thus also stimulate chronic inflammation and oxidative stress [75,76]. Therefore, sRAGEs have been investigated for their potential biomarker role in various vascular disorders, including atherosclerosis and aortopathies [24,76].

The need for exploration of the potential circulating biomarker role of sRAGEs in the prediction of presence and prognosis of aortic aneurysms had first been proposed by Sarkar et al., who described in a review article the role of the sRAGE pathway in the development of aortic disease [73]. This theory was sustained by previously published experimental studies conducted in mice, which suggested that sRAGE levels increase in the presence of thoracic aortic aneurysms and that the negative modulation of RAGE is a potential therapeutic solution towards inhibiting the formation and progression of aneurysms [50,77,78]. The AGE-sRAGE axis has been hypothesized to be involved in thoracic aortic aneurysm pathogenesis, with a significant correlation between the AGE/endogenous secretory RAGE (esRAGE) ratio and IL-2 and IL-6 release [79].

Elevated sRAGE values have been also correlated with the sole presence of BAV, not necessarily in relation to the aortic diameter [24]. On the other hand, the periodic monitorization of this biomarker might be useful in evaluating the progression of bicuspid aortic aneurysms through the NF-kB pathway [80].

In children, studies investigating the role of sRAGE in aortopathies and aortic wall impairment are missing. A pediatric study has shown that childhood sRAGE levels are physiologically higher than in adults and that, consequently, their biomarker potential in the diagnosis of mild pulmonary arterial hypertension might only be considered in adult populations [81]. Thus, their role in various pathologies cannot yet be established and requires further analysis.

### 1.4. Transforming Growth Factor Beta (TGF-β)

TGF-β includes a family of cytokines that regulates cell growth, differentiation and inflammation and is involved in vascular remodeling through ECM composition regulation [82,83]. Biological effects of TGF-β release might be dependent upon serum concentration, but its role in maintaining vascular wall integrity can no longer be contested [84,85]. Low levels of TGF-β stimulate smooth muscle cell and endothelial cell proliferation, while high levels inhibit the aforementioned physiological processes [86].

The involvement of TGF-β in ECM modeling is still controversial. Evidence has shown that TGF-β enhances collagen production, leads to fibrosis, and inhibits matrix degradation [87,88]. Moreover, TGF-β seems to play an important part in maintaining the equilibrium of the ECM content. The augmentation of TGF-β expression seems to be able to stabilize ECM remodeling by decreasing inflammatory infiltrates among the arterial wall and MMP [89]. On the other hand, TGF-β signaling pathway activation stimulates MMP-2 and MMP-9 production, which both determine matrix degradation, a key milestone in aortic aneurysm formation [90]. The activation of this pathway has been one thoroughly discussed in Marfan syndrome and is also responsible for the augmented expression of the SMAD-dependent profibrotic signaling pathway, which will lead to ECM remodeling through the promotion of collagen, elastin, fibrillin or fibronectin synthesis [91]. Thus, multiple studies have tried to determine the biomarker role of TGF-β in relation to aortic aneurysms, in light of its profibrotic characteristics and extensive ECM degradation involvement [88].

TGF-β signaling has been implied to protect against abdominal and thoracic aortic aneurysm formation, while systemic TGF-β inhibition enhances abdominal aortic aneurysm formation, whereas smooth muscle-cell specific TGF-β neutralization can increase the chances of aortic thoracic aneurysm development [92]. Furthermore, experimental TGF-β hampering in mice has stimulated aneurysm formation, as well as multiple complications associated with the disease [93]. However, contradictorily to those previous findings, the inhibition of TGF-β signaling in myeloid cells has been suggested by Hara et al. as a potential therapeutic target in patients with Marfan syndrome [94]. Similarly, an increase in serum TGF-β has been correlated with the prognosis of thoracic aortic aneurysms, as well as dissection evolvement, especially in those patients with Marfan syndrome [95,96].

Bicuspid aortic valve patients present a lower circulating TGF-β than those with TAV, probably as a result of extracellular space sequestration [97]. However, a concomitant genetic syndrome diagnosis in BAV patients has been associated with a higher circulating TGF-β than the one depicted in subjects with non-syndromic BAV or TAV [98]. A ratio between TGF-β and endoglin (ENG) has been proposed as a biomarker of BAV aortopathy [98].

Pediatric studies have mainly focused on congenital cardiac disorders, as TGF-β overexpression has been identified in populations with CHD, Marfan syndrome and aortic dilatation [99,100,101]. The elevation of TGF-β, MMP-2 and MMP-9 levels has been identified in children after surgery for tetralogy of Fallot repair (TOF) [82]. A study published by Shiina et al. also identified elevated TGF-β1 levels in patients with a repaired Fallot, in association to increases in ascending aortic diameter along with other conventional investigations, such as brachial-ankle pulse wave velocity [102].

**Figure 1:**
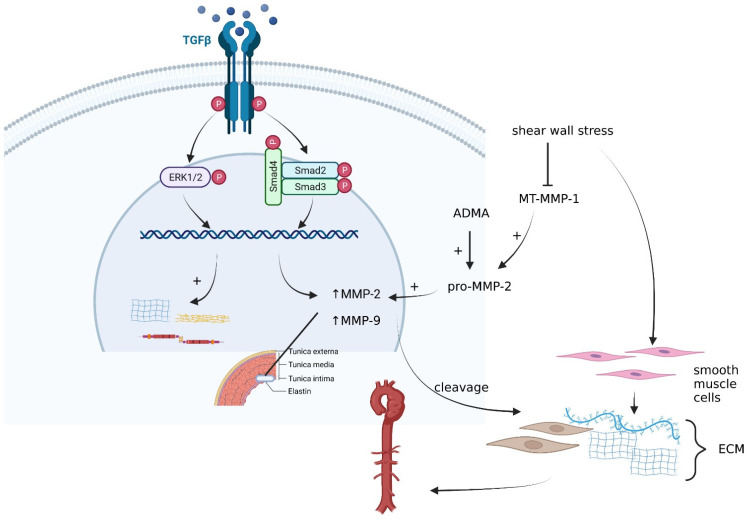
Interconnecting pathways and key effectors of aortic aneurysm development. Created with BioRender.com. Legend: The figure summarizes the consequence of TGF-β activation, which will lead to the activation of ERK1/2- and SMAD-dependent pathways, resulting in the promotion of MMP-2, MMP-9 release, which promote ECM cleavage as well as collagen, elastin and fibronectin synthesis. Shear wall stress stimulates migration of SMCs through the extracellular matrix, resulting also in MT-MMP-1 and, consequently, MMP-2’s reduced expression. ADMA activates the MMP-2 precursor, pro-MMP-2.

In conclusion, the biomarker potential of TGF-β in the prediction of aortic aneurysms is still controversial, due to multiple studies showing contradicting results in terms of expression variation. Still, TGF-β helps in maintaining the equilibrium of the arterial wall component through its influence on the ECM. The influence of TGF-β on the ECM, its effector pathways and intricate role with other molecular entities in the development of aortic aneurysms has been highlighted in Figure 1.

## 2. Future Perspectives

The genetic basis of aortopathies still requires further research, but the discovery and validation of novel molecules involved in the pathogenesis of aortic dilation and technological advances have created the premises for the depiction of their enzymatic activity, mRNA expression and analysis of single nucleotide polymorphisms’ implication. The tissular expressions of several MMPs, TIMP-1, TIMP-3, ADMA and TGF-β seem to be altered in relation to aortic dilation and aneurysms [35,58]. Single nucleotide polymorphisms of key elements such as the nitric oxide synthase-3 enzyme (Nos3), IL-6, IL-1β, IL-10, angiotensin 1 receptor and angiotensin-converting enzyme have also been regarded as potential biomarkers of aortic remodeling [56,103,104]. The main objectives of recent studies have been to identify circulating biomarkers, but a correlation between circulating and tissular biomarkers still requires clarification. Some authors have found, for example, a positive correlation between circulating TGF-β and tissular MMP-2 [98] or between circulating and tissular MMP-9 in abdominal aortic aneurysms [105], but research is still pending. The validation of non-invasive biomarkers and future investigation of particular genotypes might represent important steps towards the prognostic evaluation of incipient and evolving aortic dilatation.

## 3. Conclusions

Multiple quantifiable serum signaling pathway-associated molecules have been assessed for their potential biomarker role in the progression of aortic dilatation and prediction of aneurysm formation and associated complications. MMPs have been by far the most intensively researched, and most studies have focused on MMP-2 and MMP-9. Furthermore, most data available in the literature have analyzed candidate biomarkers in BAV, which is highly frequent among CHD. However, pediatric data is scarce, and further studies are mandated to elucidate contradicting results and identify plasmatic biomarkers, especially quantifiable at younger ages, as these can be closely followed up in the long term.

## Figures and Tables

**Table 1:** Summary of literature data sustaining biomarker role of tissular MMP-1, MMP-2, MMP-9, TIMP-1 and TIMP-2 among several aortic-related pathologies.

Type of Metalloproteinase	Expression Variation	Type of Study/Population Involved	Correlation with Conditions Studied
MMP-1	Upregulation	Case-control study, 13 patients: 8 patients with abdominal aortic aneurysm; 5 patients with normal aorta—organ donors.	Abdominal aortic aneurysm—Tamarina et al. [37]
	Upregulation—significantly among the intima	Study group—21 patients with aortic dissection; in 19 cases, expression in remote sites was assessed. Controls—10 autopsies.	Aortic dissection—Ishii et al. [46]
MMP-2	Upregulation	Experimental—organ donor tissue.	Athero-occlusive disease, and abdominal aortic aneurysm—Thompson et al. [38]
		Case-control study, 26 patients with aortic stenosis/insufficiency: 16 patients with TAV; 10 patients with BAV; 13 patients (7 patients with bicuspid aortic valve and 6 patients with tricuspid aortic valve) with aortic aneurysm/dissection; Control group—4 young cadavers, normal, tricuspid aortic valve.	BAV—Koullias et al. [42]
		Case-control study: 53 patients with BAV; 46 patients with TAV; 25 patients with no aortic abnormalities—control group.	BAV—Ikonomidis et al. [35]
		Study population: 21 patients with BAV; 16 patients with TAV.	BAV and vascular fibrilin-1 deficiency—Fedak et al. [62]
	MMP-2 deficiency	Experimental—mice.	Lack of abdominal aortic aneurysm production—Longo et al. [44]
	Upregulation—significantly among the intima	Study group—21 patients with aortic dissection; in 19 cases, expression in remote sites was assessed. Controls—10 autopsies.	Aortic dissection—Ishii et al. [46]
MMP-9	Upregulation	Case-control study, 13 patients: 8 patients with abdominal aortic aneurysm; 5 patients with normal aorta—organ donors.	Abdominal aortic aneurysm—Tamarina et al. [37]
		Case-control study, 48 patients: 40 patients with abdominal aortic aneurysm; 8 organ donors with normal infra-renal abdominal aortas.	Abdominal aortic aneurysm—Li et al. [41]
		Case-control study, 26 patients with aortic stenosis/insufficiency: Tricuspid aortic valves—16 patients; Bicuspid aortic valve—10 patients; Aortic aneurysm/dissection—13 patients (7 patients with bicuspid aortic valve and 6 patients with tricuspid aortic valve); Control group—4 young cadavers, normal, tricuspid aortic valve.	BAV, abdominal aortic aneurysm/dissection—Koullias et al. [42]
		Case—control study: 19 patients undergoing abdominal aortic aneurysm repair; 4 aortic specimens from organ donor bank.	Aortic abdominal aneurysm, particular significant association with moderate size aneurysm—McMillan et al. [48]
		Study population: 21 patients with BAV; 16 patients with TAV.	BAV and vascular fibrilin-1 deficiency—Fedak et al. [62]
	MMP-9 deficiency	Experimental—mice.	Lack of abdominal aortic aneurysm production—Longo et al. [44]
	Upregulation—significantly among the intima	Study group—21 patients with aortic dissection; in 19 cases, expression in remote sites was assessed. Controls—10 autopsies.	Aortic dissection—Ishii et al. [46]
TIMP-1	Upregulation	Experimental—organ donor tissue.	Abdominal aortic aneurysm—Thompson et al. [38]
		Case—control study, 13 patients: 8 patients with abdominal aortic aneurysm; 5 patients with normal aorta—organ donors.	Abdominal aortic aneurysm—Tamarina et al. [37]
		Case-control study, 26 patients with aortic stenosis/insufficiency: Tricuspid aortic valves—16 patients; Bicuspid aortic valve—10 patients; Aortic aneurysm/dissection—13 patients (7 patients with bicuspid aortic valve and 6 patients with tricuspid aortic valve); Control group—4 young cadavers, normal, tricuspid aortic valve.	BAV in aortic stenosis subjects—Koullias et al. [42]
	Upregulation—significantly among the intima	Study group—21 patients with aortic dissection; in 19 cases, expression in remote sites was assessed. Controls—10 autopsies.	Aortic dissection—Ishii et al. [46]
TIMP-2	Upregulation	Case—control study, 13 patients: 8 patients with abdominal aortic aneurysm; 5 patients with normal aorta—organ donors.	Abdominal aortic aneurysm—Tamarina et al. [37]
	Upregulation—significantly among the intima	Study group—21 patients with aortic dissection’ in 19 cases, expression in remote sites was assessed. Controls—10 autopsies.	Aortic dissection—Ishii et al. [46]

Legend: BAV—bicuspid aortic valve; MMP—matrix metalloproteinase; TAV—tricuspid aortic valve; TIMP—tissue inhibitor of metalloproteinase.

**Table 2:** Summary of literature data sustaining biomarker role of circulating MMP-1, MMP-2, MMP-9 and TIMP-1 among several aortic-related pathologies.

Type of Metalloproteinase	Expression Variation	Type of Study/ Population Involved	Correlation with Conditions Studied
MMP-1	Upregulation	125 patients with ascending aortic aneurysms	Ascending aortic aneurysm surgery prediction; correlation with WSS and TAWSS—Pasta et al. [40]
		Case-control study: 30 patients with acute aortic dissection; 30 healthy controls.	Acute aortic dissection—Liao et al. [49]
MMP-2	Upregulation	125 patients with ascending aortic aneurysms	Ascending aortic aneurysm surgery prediction; correlation with WSS and TAWSS—Pasta et al. [40]
		Case-control study: 30 patients with acute aortic dissection; 30 healthy controls.	Acute aortic dissection—Liao et al. [49]
MMP-9	Upregulation	Case-control study: 25 patients with chronic thoracic aortic aneurysm; 15 healthy blood donors.	Final stages of chronic thoracic aortic aneurysm—Zhang et al. [50]
		Study population (93 subjects): 37 patients with isolated severe stenotic BAV with dilated ascending aorta; 28 patients with isolated severe stenotic BAV with normal ascending aorta; 12 patients with echocardiographically normal BAV with dilated ascending aorta; 16 patients with echocardiographically normal BAV with normal ascending aorta.	Severe, isolated aortic stenosis in BAV patients—Wang Y [27]
		Pediatric case-control study (110 patients): 96 patients with VSD; 14 healthy controls.	VSD; spontaneous closure of VSD—Cheng et al. [61]
		Case-control study: 25 patients with chronic thoracic aortic aneurysm; 15 healthy blood donors.	Final stages of chronic thoracic aortic aneurysm—Zhang et al. [50]
TIMP-1	Upregulation	125 patients with ascending aortic aneurysms	Ascending aortic aneurysm surgery prediction; correlation with WSS and TAWSS—Pasta et al. [40]

Legend: BAV—bicuspid aortic valve; MMP—matrix metalloproteinase; TAV—tricuspid aortic valve; TAWSS—time average wall shear stress; TIMP—tissue inhibitor of metalloproteinase; WSS—wall shear stress.

## Data Availability

Not applicable.

## Abbreviations

ADMA	Asymmetric dimethylarginine
AGE	Advanced glycation end-product
BAV	Bicuspid aortic valve
CHD	Congenital heart disease
CT	Computed tomography
ECM	Extracellular matrix
esRAGE	Endogenous secretory RAGE
MMP	Matrix metalloproteinase
MRI	Magnetic resonance imaging
MT-MMP	Membrane-type matrix metalloproteinase
NF-κB	Nuclear-factor kappa-B
NO	Mitric oxide
Nos3	Nitric oxide synthase-3 enzyme
SMC	Smooth muscle cell
SNP	Single nucleotide polymorphisms
sRAGE	Soluble receptor for advanced glycation end-products
TAV	Tricuspid aortic valve
TAWSS	Time average wall shear stress
TGF-β	Transforming growth factor beta
TIMP	Tissue inhibitor of metalloproteinase
TLR	Toll-like receptor
TOE	Transoesophageal echocardiography
TOF	Tetralogy of Fallot
TTE	Transthoracic echocardiography
VSD	Ventricular septal defect
WSS	Wall shear stress
